# UNCP-18 is a *C. elegans* STXBP homolog that is required for full fertility

**DOI:** 10.17912/micropub.biology.002015

**Published:** 2026-05-07

**Authors:** Isabella K Jacobs, Nina Peel

**Affiliations:** 1 Department of Biology, College of New Jersey, Ewing, NJ, US

## Abstract

Exocytosis supplies the plasma membrane with lipids and proteins, and is essential for release of molecules to the cell exterior. The capture and efficient fusion of vesicles with the plasma membrane relies on SNARE and SEC1/MUNC18 (SM) proteins. The human SM protein STXBP1 is required for exocytosis of neurotransmitters, and mutation is associated with human neuronal disease. In contrast, mutations in STXBP2 and -3 primarily affect the function of non-neuronal cell types. Here we characterize the role of the *C. elegans* SM protein UNCP-18 in the germline. Endogenously tagged UNCP-18 is broadly expressed and primarily localized to the plasma membrane, consistent with a role in exocytosis. Loss of UNCP-18 led to reduced brood size which can be partially rescued by mating. Additional germline defects are evident, however, suggesting a role beyond the sperm. Our results demonstrate that UNCP-18 plays non-neuronal roles and suggest that *C. elegans* might provide a useful model for understanding the non-neuronal functions of SM proteins.

**Figure 1. uncp-18(tm530) mutants have a reduced brood size due to reduced sperm number and germline defects:** A) Brood size and B) viability of WT and *uncp-18 ( tm530 )* hermaphrodites at indicated culture temperatures. C) Brood size of hermaphrodites of the stated genotype when mated with *him-5* males. D) Brood size of *fog-1* females mated with males of the indicated genotypes. E) Summary of relative sperm count in 1day old adult hermaphrodite spermathecae, and correlation with germline phenotypes. *uncp-18* heterozygous worms were used as a control. F&G) UNCP-18 ::mNG-expressing worms. F) Plasma membrane localization in the germline, G) A two-cell embryo at telophase. Arrows indicate likely vesicular association. Arrowheads indicate perinuclear localization. ** p<0.01; * p<0.05, Student's t test.

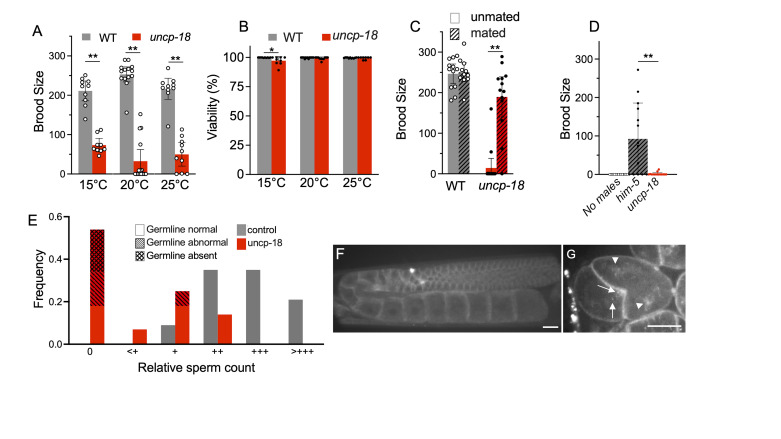



## Description

Exocytosis is important for delivery of proteins and lipids to the cell surface, and for the release of cargo to the cell exterior. Multiple steps in exocytosis are mediated by conserved SNARE proteins and by SEC1/MUNC18 (SM) proteins. Once vesicles arrive at the membrane, vSNAREs on the vesicle membrane and tSNARES on the plasma membrane assemble into a trans-SNARE complex which facilitates vesicle docking. The SM-family proteins act as chaperones to ensure the fast and accurate assembly of the trans-SNARE complex, aiding efficient membrane fusion.

Humans have three SM family proteins that regulate exocytosis, and their dysfunction is associated with disease. STXBP1 mutations cause early infantile epileptic encephalopathy (EIEE) (Saitsu et al., 2010); STXBP2 mutations cause familial hemophagocytic lymphohistiocytosis 5 (F-HLH5) (Côte et al., 2009; Spessott et al., 2017); and STXBP3 mutations cause very early onset inflammatory bowel disease (VEOIBD) (Ouahed et al., 2021). In each case, disease phenotypes arise due to impaired exocytosis. Neurons require STXBP1 for synaptic vesicle release. Immune cells use STXBP2 for cytotoxic granule extrusion. And gut epithelial cells require STXPB2/3 for apical cargo delivery (Ouahed et al., 2021; Vogel et al., 2017).

The *C. elegans* genome encodes two SM family proteins, UNC-18 and UNCP-18 (Boeglin et al., 2023). Of these, UNC-18 is important for synaptic transmission, and its role in neurotransmitter release suggests a functional similarity to STXBP1 (Hosono et al., 1992). In comparison, the role of the paralog UNCP-18 is less well studied. At the outset of this project uncp-18 was uncharacterized, but a recent paper described a role for UNCP-18 in neurons where it functions redundantly with UNC-18 (Boeglin et al., 2023). Given that STXBP2 and -3 play important non-neuronal roles, we sought to characterize UNCP-18 functions in non-neuronal contexts focusing on the germline.

We obtained the *uncp-18 ( tm530 )* mutant allele, a 336bp deletion that is predicted to cause an in-frame 48 amino acid deletion (p.310-358) in a region of the protein that is highly conserved with human STXBP proteins (Boeglin et al., 2023; Harris et al. 2020). Although viable, the strain was difficult to maintain due to low numbers of offspring. Because of this, we crossed the mutation to the marked *dpy-9 ( tm9713 ) kvs-5 ( tmIs1245 )* chromosome, and subsequently maintained it as a balanced strain, selecting *uncp-18 ( tm530 )* homozygotes for each analysis. A brood size analysis showed that homozygous *uncp-18* mutants had a striking decrease in fertility ( Figure 1A ), with brood size reaching a maximum of ~30% of wild-type. This phenotype was evident across all assayed temperatures indicating that the phenotype was not temperature-sensitive. In contrast, we found that embryonic viability was similar to wild type ( Figure 1B ).

Since *C. elegans* are self-fertilizing hermaphrodites, we reasoned that the reduced brood size could result from either sperm or oocyte defects. To differentiate between these possibilities, we mated control males with *uncp-18* hermaphrodites. Mating significantly increased the brood size of the *uncp-18* hermaphrodites ( Figure 1C ), suggesting that the reduction in fertility was at least partially attributable to a sperm defect. In parallel, we tested sperm function in *uncp-18* males by mating them with feminized worms. While control *him-5* males produced upward of 100 offspring, most *uncp-18* males produced none. Only three males sired offspring, and in each case fewer than 13 embryos were produced ( Figure 1D ). This reduced fertility of *uncp-18* males points to sperm deficiency as a major contributor to the reduced brood size phenotype. Our results largely concur with a recent analysis of an independently derived *uncp-18* null allele (Boeglin et al., 2023).

To investigate the sperm defect further, we crossed a spe-11-promoter-driven fluorescent transgene (which is expressed specifically in sperm) with *uncp-18* worms. This sperm-marked strain allowed us to score relative sperm abundance in one day old hermaphrodites. We found that more than 50% of *uncp-18* worms completely lacked sperm ( Figure 1E ). Of these, approximately one third possessed structurally intact germlines, one third had germlines that appeared abnormal, and one third lacked any discernible germline, indicating the sperm deficiency has pleiotropic causes. The remaining ~40% of *uncp-18* worms which did possess sperm tended to show a lower sperm abundance than control worms (Fig 1E). These data suggest that UNCP-18 contributes to fertility at multiple levels, including in germline development and in germ cell production.

Finally, to assess whether, similar to homologs, UNCP-18 plays a role in exocytosis, we integrated a neon green tag into the endogenous locus. The fusion protein was widely expressed including in the germline and embryos. We found that UNCP-18 ::mNG localized at the plasma membrane throughout the germline ( Figure 1F ). It also shows a strong signal between adjacent cells in the two-cell embryo ( Figure 1G ), and can be seen in small cytoplasmic particles that move towards the membrane at cytokinesis (arrows). In addition, a particulate localization in close proximity to the nucleus can be observed (arrowheads), suggestive of localization to internal membranes. This localization is consistent with UNCP-18 contributing to vesicle trafficking and exocytosis.

Given its homology and subcellular localization, it is likely that UNCP-18 functions in exocytosis in *C. elegans .* Our data suggest, however, that it is not essential in the embryo *.* At fertilization the regulated exocytosis of cortical granules is required for eggshell formation, and failure to establish this permeability barrier leads to embryonic lethality (Sato et al., 2008). In addition, exocytosis is essential later in embryogenesis with impairment of secretory pathways leading to defects in cuticle and pharynx formation (Roberts et al., 2003). Although our expression/localization data show that UNCP-18 is present in the embryo, we found that *uncp-18* mutant embryos were viable and survived at levels comparable to wild type. These embryos were produced by mutant hermaphrodites so they lacked any maternal UNCP-18 contribution. The observed viability therefore suggests that UNCP-18 is not essential for exocytosis in the embryo. It has been reported that *unc-18 ( e81 ); uncp-18 ( syb6377 )* double mutants are embryonic lethal, indicating that UNCP-18 functions redundantly with UNC-18 in the embryo (Boeglin et al., 2023).

In contrast, loss of UNCP-18 leads to a range of germline phenotypes, from a reduction in gamete number to complete germline loss. A majority of hermaphrodites have partial rescue of brood size upon mating ( Figure 1C ), indicating that a sperm deficit contributes to the brood size phenotype. In addition to reduced sperm number, however, structural defects in the germline were also observed. Our data suggest, therefore, that UNCP-18 is required for both germline development/maintenance, and germ cell production. The underlying basis for this germline defect remains unknown.

In summary, we have characterized germline phenotypes associated with loss of UNCP-18 . Our data show that UNCP-18 is membrane-associated, consistent with a role in exocytosis, and that UNCP-18 is required for full fertility. Determining the exact roles UNCP-18 plays in the germline will require further investigation. Importantly, our initial analyses have identified *C. elegans* as a model for understanding the non-neuronal functions of STXBP proteins with the potential to illuminate the etiology of associated diseases.

## Methods

*C. elegans* strains were cultured on MYOB plates seeded with OP50 bacteria at 20°C unless stated otherwise. For brood size and viability assays, single L4 hermaphrodites were transferred onto 35mm plates. After 24 hours, each worm was transferred to a new plate, and this was repeated until embryo production had ceased. Each plate was scored 24h after adults were removed, and the number of viable worms and embryos was recorded. Brood size was calculated by summing viable worms and unhatched embryos. Viability was calculated as viable worms/broodsize. The ability of mating to rescue *uncp-18* brood size was tested by putting four *him-5* males and one L4 hermaphrodite onto 35mm plates. After 24 hours, worms were transferred to a new plate, and this was repeated until embryo production ceased. Each plate was scored 24h after adults were removed and the number of viable worms and embryos was recorded. To test fertility of *uncp-18* males, *fog-1* females were generated by moving L4 hermaphrodites to 25°C whereby all offspring are feminized. One male and one *fog-1* female were transferred onto 35mm plates and after 24 hours worms were transferred to a new plate. This was repeated for 3days. Each plate was scored 24h after embryos were laid, and the number of viable worms and embryos was recorded. The *uncp-18 ::mNG* strain was made by CRISPR-mediated integration of the neonGreen ORF at the endogenous uncp-1 locus (SUNY Biotech). For imaging, the worms were anesthetized in 20mM sodium azide, mounted on a 2% agarose pad and viewed on a Diskovery spinning disk confocal system (Andor) mounted on a Nikon Eclipse Ti microscope with a 60×/1.4 NA objective. Images were captured using an ImagE Mx2 EM-CCD camera (Hamamatsu Photonics, Shizuoka, Japan). Metamorph software was used for Image capture.

## Reagents

**Table:** 

Strain	Genotype	Available from
N2	*Wild type*	CGC
DR466	*him-5 ( e1490 ) V *	CGC
DE90	*oxIs318 II; unc-119 ( ed3 or e2498 ) ruIs32 III; ddIs6 V; dnIs17*	CGC (Johnston et al., 2010)
FX30269	*dpy-9 ( tm9713 ) kvs-5 ( tmIs1245 ) IV *	CGC (Dejima et al., 2018)
NIN143	*oxIs318 [spe-11p::mCherry::histone + unc-119 (+)] II ; uncp-18 ( tm530 )/ dpy-9 ( tm9713 ) kvs-5 ( tmIs1245 ) IV; him-5 ( e1490 ) V *	authors
NIN114	*uncp-18 ( tm530 )/ dpy-9 ( tm9713 ) kvs-5 ( tmIs1245 ) IV *	authors
NIN129	*uncp-18 ( tm530 )/ dpy-9 ( tm9713 ) kvs-5 ( tmIs1245 ) IV; him-5 ( e1490 ) V *	authors
PHX8051	*uncp-18 ( syb8051 [ uncp-18 ::mNG]) IV *	authors; SUNY Biotech
JK560	*fog-1 ( q253 ) I *	CGC
